# TrED: the *Trichophyton rubrum *Expression Database

**DOI:** 10.1186/1471-2164-8-250

**Published:** 2007-07-25

**Authors:** Jian Yang, Lihong Chen, Lingling Wang, Wenliang Zhang, Tao Liu, Qi Jin

**Affiliations:** 1 State Key Laboratory for Molecular Virology and Genetic Engineering, National Institute for Viral Disease Control and Prevention, Chinese Center for Disease Control and Prevention, Beijing 100176, China; 2 Institute of Pathogen Biology, Chinese Academy of Medical Sciences, Beijing 100730, China

## Abstract

**Background:**

*Trichophyton rubrum *is the most common dermatophyte species and the most frequent cause of fungal skin infections in humans worldwide. It's a major concern because feet and nail infections caused by this organism is extremely difficult to cure. A large set of expression data including expressed sequence tags (ESTs) and transcriptional profiles of this important fungal pathogen are now available. Careful analysis of these data can give valuable information about potential virulence factors, antigens and novel metabolic pathways. We intend to create an integrated database TrED to facilitate the study of dermatophytes, and enhance the development of effective diagnostic and treatment strategies.

**Description:**

All publicly available ESTs and expression profiles of *T. rubrum *during conidial germination in time-course experiments and challenged with antifungal agents are deposited in the database. In addition, comparative genomics hybridization results of 22 dermatophytic fungi strains from three genera, *Trichophyton*, *Microsporum *and *Epidermophyton*, are also included. ESTs are clustered and assembled to elongate the sequence length and abate redundancy. TrED provides functional analysis based on GenBank, Pfam, and KOG databases, along with KEGG pathway and GO vocabulary. It is integrated with a suite of custom web-based tools that facilitate querying and retrieving various EST properties, visualization and comparison of transcriptional profiles, and sequence-similarity searching by BLAST.

**Conclusion:**

TrED is built upon a relational database, with a web interface offering analytic functions, to provide integrated access to various expression data of *T. rubrum *and comparative results of dermatophytes. It is devoted to be a comprehensive resource and platform to assist functional genomic studies in dermatophytes. TrED is available from URL: .

## Background

Dermatophytes are fungi that can cause superficial infections of the skin, hair, and nails. They are the most common agents of fungal infections worldwide and impact millions of individuals annually [1,2]. Because of the severity and longevity of the disease and its refractivity to therapy, dermatophyte infections cause tremendous pain and account for significant costs to society. The dermatophytic fungi include numerous species of fungi which belong to the following three genera; *Epidermophyton*, *Microsporum *and *Trichophyton*. *T. rubrum *is the most commonly observed dermatophyte worldwide and especially dominant in onychomycosis with a prevalence of approximately 80% [3].

However, very few biochemical identification procedures are available for dermatophytes; thus, diagnosis of dermatophyte infections is based on the gross and microscopic morphology of the colony. Fungal culture, which can be useful to confirm the diagnosis and treatment, would take two to four weeks and pleomorphic growth can lead to misidentification. Molecular epidemiology of an outbreak of fungal infections is also not possible due to the lack of molecular methods [4]. Though a number of pathogenic fugal genomes were determined within the last decade, to date, however, no dermatophyte genome is publicly available yet. Moreover, dermatophytic sequences are surprisingly poor-represented in public databases; besides the recently released ESTs by our group and others (see below) only 524 nucleotide sequences (including a majority of ribosomal RNA sequences for phylogenetic studies) were found in GenBank for all three genera of dermatophytes by April 2007. Therefore the dearth of publicly available genomic data is a major barrier to the current biomedical research of dermatophytes.

Fortunately, a set of five dermatophytes including *T. rubrum *were recently proposed for genome sequencing by the National Human Genome Research Institute [5]. It would provide key insights into the pathogenic life style of dermatophytes and boost the development of new diagnostics, therapies, and vaccines. However, in the absence of complete genomic sequences, single pass, partial sequencing of either 3' or 5' ends of complementary DNA (cDNA) clones to generate a set of expressed sequence tags (ESTs), offers a highly cost-effective strategy of accessing and identifying gene inventories. The availability of EST datasets is also important to future genome annotation and gene expression analysis.

Recently, our group reported a sequencing program of over thirty thousands ESTs derived from ten different stages of *T. rubrum *life cycle, which represented a first significant step towards the comprehensive description of cellular functions involved in *T. rubrum *biology [6]. Since microarrays are widely recognized as a significant technological advance providing transcriptome expression patterns, we further constructed *T. rubrum *cDNA microarrays from the EST clones and applied them in the following studies. One is tracing changes of genes expression during *T. rubrum *conidial germination in time-course experiments to reveal molecular mechanisms in developmental stage at the cell level [7]. The other is transcriptional profiles studies of *T. rubrum *response to several antifungal agents to make clear the mechanism of drug actions in this pathogenic fungus [8,9]. And the third one is a comparative genomic hybridization (CGH) analysis to assess gene variation among different dermatophytes, which may yield some insights into the host-specificity and pathogenesis in dermatophytic fungi (our unpublished data).

The enormous information from *T. rubrum *needs to be well organized and presented for researchers focusing on dermatophytes. TrED is therefore developed to the accurate interpretation and incisive exploitation of massive datasets. The database integrates of genetic, transcriptomic and metabolomic data of *T. rubrum *as well as comparative genomics results of dermatophytes. As a result, TrED provides a wealth of invaluable information about the evolution, life cycles, cell biology, and virulence of dermatophytes.

## Construction and content

### Data sources

TrED currently collects the following three types of data: (i) EST sequences of *T. rubrum*, (ii) transcriptional profiles of *T. rubrum *and (iii) CGH results of 22 dermatophytes.

The raw EST data in TrED include all publicly available *T. rubrum *EST sequences as well as a set of our newly determined ESTs derived from *T. rubrum *mycelia grown under conditions mimicking virulence. To suppress the potential sequencing errors that inherent in single-pass reads, all ESTs sequenced by our group were reevaluated from the original chromatograms by setting the Phred quality score cutoff to 20, which means less than one expected error per 100 base pairs (bp). Public data deposited by other contributors were directly retrieved from dbEST [10]. Redundant and obsolete records were removed.

Transcriptional profiles of *T. rubrum *response to different antifungal agents and its expression data during conidial germination reported previously were available from Gene Expression Omnibus (GEO) [11]. Data from a newly performed CGH study of 22 dermatophyte strains were also integrated into TrED.

### Analytical methods

All ESTs were screened to remove contaminating sequences, including restriction site, adaptor, cloning vector, poly(A/T) tail and bacterial sequences by the trimming script SeqClean [12] with NCBI's UniVec as filtering database. ESTs with remain length <50 bp were excluded from further analysis.

Since ESTs are typically partial, redundant and error-prone, the TGICL software [13] was used to form clusters of similar ESTs with the following criteria: ≥95% identity of overlaps with ≥40 bp in length and mismatched overhangs <20 bp. The ESTs comprising each cluster were assembled using CAP3 [14] to produce longer and more reliable consensus sequences (i.e. contigs). ESTs that can't be clustered or assembled with others were kept as singletons. Only contigs or singletons with valid length ≥100 bp were deposited into the database. There are 10,224 different assembled *T. rubrum *sequences (unisequences) composed of 4,566 contigs and 5,658 singletons in the current release of TrED.

ESTScan2 was used to detect possible coding regions in all unisequences [15]. Since available *Trichophyton *coding sequences (CDS) were limited from public domains as mentioned above, we used a combination of CDSs from genomes of two relatives, *Coccidioides immitis *and *Aspergillus fumigatus*, as the training dataset for building hidden Markov models. The 944 ESTs without a detectable coding region are largely (81.4%) singletons and short in size, which are probably mostly made of untranslated region.

The predicted peptides were then used for conserved protein families search in Pfam database [16] by HMMER software [17], and sequence-similarity search by BLASTP in databases of Gene Ontology (GO) [18], eukaryotic orthologous groups (KOG) [19] and KEGG [20], respectively. The nucleotide sequences were also sent to similarity search by BLASTX against the non-redundant protein database of GenBank (NR). All high-throughput analytic approaches mentioned above were facilitated by using local version of databases and programs.

The nomenclature from MEROPS database was adopted for the classification of putative proteases found in *T. rubrum *[21]. Possible transmembrane domains were identified by TMHMM2.0 [22].

### Database implementation

TrED is built on a RedHat Linux 9.0 operation system and the data are stored as a MySQL relational database that is accessible directly through an Apache web server. The Perl programming language and some common modules, such as DBI, GD and CGI, are used to generate interactive web pages for the query interfaces. A revision of the KEGG pathway map-viewer [23] is employed for graphic representations of metabolic pathways based on user-defined similarity settings (see below). A local WWW-BLAST program is integrated into TrED to allow users performing sequence-similarity search against all currently available sequences of dermatophytes (nucleic acid or amino acid) using the BLAST algorithm.

The weekly update of datasets for dermatophyte BLAST is accomplished through automated downloading new data from GenBank by BioPerl scripts. A bimonthly execution of BLASTX comparison between all unisequences in TrED and the current NR database is scheduled on a background Linux-cluster, and the results will be transferred and imported into the database in a semi-automated fashion by a series of Perl scripts.

## Utility and discussion

### Database overview

The TrED database supports the following basic tasks: (i) browsing by listing of cDNA libraries, assembled unisequences or related metabolic pathways, (ii) querying based on accession numbers, clone names, functional classifications or protein properties as well as sequence-similarity searching by BLAST, (iii) visualizing and comparing transcription profiles and CGH data and (iv) downloading the raw data and analysis results.

TrED integrated several different types of expression data for *T. rubrum *including EST sequences and transcriptional profiles. Though most of them are also available from other public databases, such as dbEST and GEO, they keep only the raw data with virtually no biological content. However, researchers who interested in further data mining prefer resources with comprehensive biological information rather than raw data depositories. TrED described here are essential for this goal. It provides additional information for individual ESTs including assembly structure, predicted peptide, Gene Ontology associations, and multiple sources of comparison to infer functional annotation. Moreover, different types of data are interconnected within the database, which is very convenient for further interpretations.

*T. rubrum *is a model organism for dermatophytes research and most data in TrED were directly derived from this organism. But the CGH results of 22 dermatophyte strains by the *T. rubrum *cDNA microarrays were also integrated into the database. It actually extends the contents of TrED to make it a valuable resource not only for *T. rubrum *study but also for studies of other dermatophytes. We further constructed a dermatophyte BLAST tool to facilitate the future researches in pathogenic superficial fungi (see below for details).

### Comprehensive and configurable user interface

In the TrED database, individual EST sequences are clustered and assembled to produce a set of more complete and reliable consensus sequences (i.e. unisequences) representative of putative genes. A typical page for display each of the unisequence contains the following information:

• Basic features including sequence length, G+C content and number of related ESTs;

• Clickable schematic view of assembly structure with the component ESTs color-coded by clone libraries (for contigs only);

• Biologist curated tentative annotation for the unisequence (if assigned);

• Functional classifications including Gene Ontology associations, KEGG Ontology (KO) classes and KOG clusters assigned (if any);

• Sequence-similarity based prediction of enzyme nomenclature (EC numbers) and possible metabolic pathways involved (if any);

• Peptidase family by the nomenclature from MEROPS database (for detectable protease only);

• Graphical representation of potential Pfam domains and putative transmembrane regions in the sequence (if any);

• Comparison table of its transcriptional responses to antifungal agents (if available);

• Plot view of its expression during conidial germination in time-series (if available);

• CGH results of 22 dermatophyte strains (if available);

• Unisequence with clickable link for download in FASTA format;

• Nucleic acid sequence of the potential coding region identified by ESTScan2 (if detectable);

• Predicted peptides that conceptually translated by ESTScan2 (if detectable);

Most of the above mentioned components of the webpage provide clickable inter links to other function pages within TrED, as well as direct links to related pages in other valuable public resources, such as GenBank, Gene Ontology, KEGG, KOG, Pfam and MEROPS. By combining various aspects of information with the ability to access different web repositories, TrED brings researchers a synergy of dynamic resources publicly available over the internet.

However, the convenience of retrieving comprehensive information from a single page is disadvantaged by requiring more network resources, which could be a serious obstacle to users who have limited local net speed. To alleviate the problem, we setup a user-friendly configure menu for users to fully customize the content to be displayed in the page. It's also amenable to researchers who have specialized interests in particular aspects of the data.

Another configurable feature for TrED users is the ability to customize similarity cutoffs for advanced data mining. Sequence-similarity based function interpretation is still the major annotation method widely used nowadays. However, the stringency of the homologous match that defines a biological function is not a constant applicable to all cases. A stringent criterion tend to lead to less information, while a loose one may result in vast of spurious matches. So predefined similarity cutoffs could be arbitrary to some researchers. The customized cutoff values take effect to all kinds of sequence-similarity based analysis in TrED, such as GO terms assignment and metabolic pathway reconstruction.

### Visualization and comparison of transcriptional profiles and CGH results

The microarray page available from TrED menu provides a platform for specialized explorations among all types of microarray-based data. The expression variations of genes during the *T. rubrum *conidial germination can be graphically represented in batch by choosing a dataset from the analysis menu. CGH results performed on 22 superficial fungal strains can be easily browsed in a tabular style upon request. Moreover, in order to facilitate further comparative analysis on the CGH data, an auto filter was set up to offer users the potential to rapidly examine commonly shared or lost genes within each genus.

Currently, transcriptional profiles of *T. rubrum *response to three antifungal agents are integrated into TrED, including ketoconazole, amphotericin B and a novel synthetic fatty acid synthase inhibitor PHS11A [8,9]. These data portray how variations in the transcription levels of particular genes related to mechanisms for drug sensitivity and resistance. TrED provides an easy interface to view and compare different gene expression and drug activity patterns. More data of all dermatophytes are expected to be involved in future update when available. This is the first attempt to integrate large gene expression database and drug discovery screen for dermatophytes.

### Search the database and dermatophyte BLAST service

TrED provides a suite of web-based tools that allow users to query and extract information from the database: (i) text based search, (ii) annotation based function category enumeration and (iii) BLAST based sequence-similarity search.

The text based database interrogation enables extracting the current tentative annotations using any querying keywords. A single entry, that is instantly familiar to users of other internet search engines, is offered for alternative query words, separated by blanks, or complex phrases enclosed by double quotation marks. The database is also searchable by using EST accession number, clone name, internal ID or a combination thereof.

The tentative annotation for each unisequence in TrED was manually assigned by biologists based on the sequence similarities among available resources, such as GenBank and Pfam. But potential false annotations are always unavoidable and they might mislead users in some cases. The extended text search engine was designed to amend this gap by providing users the ability to query the original top 100 BLASTX hits in NR database directly. As the BLASTX comparison results are automated update bimonthly, the extended search engine is particularly pertinent to active researchers by offering the most current information with potential clues for further deciphering the biology of *T. rubrum*.

Enumeration genes possibly related to given function category provides a simple way to retrieve specialized information for researchers focused on particular areas of *T. rubrum *biology. Functional classifications based on controlled vocabulary defined by GO, KOG cluster and KEGG ontology were currently adopted in TrED. The querying results, which depend on customized similarity settings (see above), are displayed in an explicit table with each hit represented by a row. Furthermore, each listed row in the output table provides inner link to the individual unisequence page as well as outer links to related public resources.

BLAST algorithm has been widely used for fast sequence-similarity searches in numerous databases. By adopting a web-based BLAST server TrED allows users to perform sequence comparison against all sequences in the database. Moreover, to facilitate the genomic research on dermatophytes, we developed a dermatophyte BLAST tool in TrED. While sequence comparisons with common databases, such as GenBank, have been invaluable for gaining a broad understanding of genomes, single gene comparisons across the relative species are often useful to researchers focused on particular areas of biology [24]. The dermatophyte BLAST service is designed as a complement and specialized subclass of the Fungal BLAST tool [24], and tailored for researchers who concentrate on pathogenic superficial fungi. The sequence datasets include all available dermatophyte sequences, both nucleic acid and amino acid, retrieved from GenBank along with ESTs, unisequences and predicted peptides from TrED. To reflect the most current sequence data available from constantly changing public databases, an automated weekly updating scheme was implemented for the dermatophyte BLAST server.

### Analyses by TrED

Perhaps one of the most characteristic features of the TrED user interface is the assignment of unisequences to various functional and structural categories, which allows users to focus on specific subset of the tremendous data.

Fungal factors contributing to virulence include antigenic variability, the presence of fungal adhesions, effective iron acquisition systems, etc. Most of these molecules are associated with the cell surface. So it's valuable to estimate the fraction of integral membrane proteins in the *T. rubrum *genome. From the current data, 1,520 (14.9%) unisequences occupy detectable transmembrane regions, including transporters, receptors, channels, sensory transducer and pumps as targets for drugs. But 67.2% of them are hypothetical proteins or orphan sequences, their real biological functions and medical significance require further investigations.

A biological characteristic of dermatophytes is their ability to invade keratinized tissues, so it is evident that secreted proteases are suspected pivotal virulence determinants. Each putative protease found in *T. rubrum *is classified to related peptidase family by nomenclatures from the MEROPS database [21]. Most of the proteases identified in *T. rubrum *belong to metallo and serine protease family and 83.9% have homologs in *A. fumigatus*. Further investigations on protease substrate specificities will improve our understanding of their functions and contributions to virulence. Furthermore, the CGH results of these proteases could help to explain the relative specificities of different dermatophytes in causing different types of dermatophytosis.

We used Pfam database to search *T. rubrum *unisequences for functional domains and other known sequence motifs. 3,448 (33.7%) had one or more Pfam hits with an hmmer E-value cutoff of 1. *T. rubrum *shares three of its top ten Pfam domains (major facilitator superfamily, WD domain, and protein kinase domain) with *C. immitis *[25] and *A. fumigatus *[26].

### Future prospects

Future TrED development will be dedicated to offer a platform for comparative genomics studies of dermatophytes. It will integrate sequence, expression and other data associated with dermatophytes. As with the ongoing efforts for genome sequencing of *T. rubrum*, certain aspects of the results will change. We will update the results described here on a regular basis and contribute to the community effort.

## Conclusion

The enormous influx of information from genome sequencing projects is revolutionizing the science of fungal pathogenesis. This ranges from understanding the most basic aspects of gene content and genome organization, to elucidating the mechanisms of host-pathogen interaction and the development of new diagnostic techniques and vaccines. With the release of the large set of expression data of *T. rubrum*, and the integrated database TrED, significant progress shall be made in unraveling the intriguing biology of this medically important fungal pathogen of humans and its mechanisms of virulence. Gaps in our knowledge will be filled by a combination of comparative and functional genomics, including techniques such as transcriptomics, bioinformatics, and proteomics.

## Availability and requirements

The database is available at  suitable for most graphical web browser. The users' browser need to enable cookie (which is supported by all modern browsers) for some database utilities, such as customized similarity setting and batch query process.

## Authors' contributions

LC designed the website, participated in data evaluation and drafted the manuscript. JY constructed the database, performed the computational analysis and revised the manuscript. LW, WZ and TL contributed data to the database. QJ conceived of the study and supervised the work. All authors read and approved the final manuscript.

## References

[B1] Weitzman I, Summerbell RC (1995). The dermatophytes. Clin Microbiol Rev.

[B2] Hainer BL (2003). Dermatophyte infections. Am Fam Physician.

[B3] Evans EG (1998). Causative pathogens in onychomycosis and the possibility of treatment resistance: a review. J Am Acad Dermatol.

[B4] Kac G (2000). Molecular approaches to the study of dermatophytes. Med Mycol.

[B5] (2006). 2006 Release: NHGRI Announces Latest Sequencing Targets. http://www.genome.gov/19517271.

[B6] Wang L, Ma L, Leng W, Liu T, Yu L, Yang J, Yang L, Zhang W, Zhang Q, Dong J, Xue Y, Zhu Y, Xu X, Wan Z, Ding G, Yu F, Tu K, Li Y, Li R, Shen Y, Jin Q (2006). Analysis of the dermatophyte Trichophyton rubrum expressed sequence tags. BMC Genomics.

[B7] Liu T, Zhang Q, Wang L, Yu L, Leng W, Yang J, Chen L, Peng J, Ma L, Dong J, Xu X, Xue Y, Zhu Y, Zhang W, Yang L, Li W, Sun L, Wan Z, Ding G, Yu F, Tu K, Qian Z, Li R, Shen Y, Li Y, Jin Q (2007). The use of global transcriptional analysis to reveal the biological and cellular events involved in distinct development phases of Trichophyton rubrum conidial germination. BMC Genomics.

[B8] Yu L, Zhang W, Wang L, Yang J, Liu T, Peng J, Leng W, Chen L, Li R, Jin Q (2007). Transcriptional Profiles of the Response to Ketoconazole and Amphotericin B in Trichophyton rubrum. Antimicrob Agents Chemother.

[B9] Zhang W, Yu L, Leng W, Wang X, Wang L, Deng X, Yang J, Liu T, Peng J, Wang J, Li S, Jin Q (2007). cDNA microarray analysis of the expression profiles of Trichophyton rubrum in response to novel synthetic fatty acid synthase inhibitor PHS11A. Fungal Genet Biol.

[B10] (2007). Expressed Sequence Tags database. http://www.ncbi.nlm.nih.gov/dbEST/.

[B11] Barrett T, Troup DB, Wilhite SE, Ledoux P, Rudnev D, Evangelista C, Kim IF, Soboleva A, Tomashevsky M, Edgar R (2007). NCBI GEO: mining tens of millions of expression profiles--database and tools update. Nucleic Acids Res.

[B12] (2007). DFCI Gene Indices Software Tools. http://compbio.dfci.harvard.edu/tgi/software/.

[B13] Pertea G, Huang X, Liang F, Antonescu V, Sultana R, Karamycheva S, Lee Y, White J, Cheung F, Parvizi B, Tsai J, Quackenbush J (2003). TIGR Gene Indices clustering tools (TGICL): a software system for fast clustering of large EST datasets. Bioinformatics.

[B14] Huang X, Madan A (1999). CAP3: A DNA sequence assembly program. Genome Res.

[B15] Lottaz C, Iseli C, Jongeneel CV, Bucher P (2003). Modeling sequencing errors by combining Hidden Markov models. Bioinformatics.

[B16] Finn RD, Mistry J, Schuster-Bockler B, Griffiths-Jones S, Hollich V, Lassmann T, Moxon S, Marshall M, Khanna A, Durbin R, Eddy SR, Sonnhammer EL, Bateman A (2006). Pfam: clans, web tools and services. Nucleic Acids Res.

[B17] Eddy SR (1998). Profile hidden Markov models. Bioinformatics.

[B18] Gene Ontology Consortium (2006). The Gene Ontology (GO) project in 2006. Nucleic Acids Res.

[B19] Tatusov RL, Fedorova ND, Jackson JD, Jacobs AR, Kiryutin B, Koonin EV, Krylov DM, Mazumder R, Mekhedov SL, Nikolskaya AN, Rao BS, Smirnov S, Sverdlov AV, Vasudevan S, Wolf YI, Yin JJ, Natale DA (2003). The COG database: an updated version includes eukaryotes. BMC Bioinformatics.

[B20] Kanehisa M, Goto S, Hattori M, Aoki-Kinoshita KF, Itoh M, Kawashima S, Katayama T, Araki M, Hirakawa M (2006). From genomics to chemical genomics: new developments in KEGG. Nucleic Acids Res.

[B21] Rawlings ND, Morton FR, Barrett AJ (2006). MEROPS: the peptidase database. Nucleic Acids Res.

[B22] Krogh A, Larsson B, von Heijne G, Sonnhammer EL (2001). Predicting transmembrane protein topology with a hidden Markov model: application to complete genomes. J Mol Biol.

[B23] Yang J, Chen L, Yu J, Sun L, Jin Q (2006). ShiBASE: an integrated database for comparative genomics of Shigella. Nucleic Acids Res.

[B24] Balakrishnan R, Christie KR, Costanzo MC, Dolinski K, Dwight SS, Engel SR, Fisk DG, Hirschman JE, Hong EL, Nash R, Oughtred R, Skrzypek M, Theesfeld CL, Binkley G, Dong Q, Lane C, Sethuraman A, Weng S, Botstein D, Cherry JM (2005). Fungal BLAST and Model Organism BLASTP Best Hits: new comparison resources at the Saccharomyces Genome Database (SGD). Nucleic Acids Res.

[B25] Broad Institute of Harvard and MIT (2007). Coccidioides immitis Sequencing Project. http://www.broad.mit.edu/.

[B26] Riley ML, Schmidt T, Wagner C, Mewes HW, Frishman D (2005). The PEDANT genome database in 2005. Nucleic Acids Res.

